# Supplementary material for: Proton-assisted calcium-ion storage in aromatic organic molecular crystal with coplanar stacked structure
Source: Nat Commun. 2021 Apr 23;12:2400. doi: 10.1038/s41467-021-22698-9 (PMC8065044; doi:10.1038/s41467-021-22698-9)
Supplement: Supplementary file 1 — Supplementary Information [file 41467_2021_22698_MOESM1_ESM.pdf]

Supplementary Information

Proton-assisted Calcium-Ion Storage in Aromatic Organic Molecular Crystal with Coplanar Stacked Structure

Cuiping Han,[1] Hongfei Li[2,*] Yu Li,[3] Jiaxiong Zhu,[2] Chunyi Zhi,[1,*]

[1] *Department of Materials Science and Engineering, City University of Hong Kong, 83 Tat Chee Avenue, Kowloon, Hong Kong 999077, China.*

[2] *Songshan Lake Materials Laboratory, Dongguan, Guangdong, 523808, China.*

[3] *College of Materials Science and Engineering, and Shenzhen Key Laboratory of Special Functional Materials, Shenzhen University, Shenzhen, 518060, China.*

E-mail: lihf@sslab.org.cn (H. Li), cy.zhi@cityu.edu.hk (C. Zhi).

Supplementary Methods

Preparation of $KCoFe(CN)_6 \cdot xH_2O$ cathode material

The Prussian blue analogues (i.e., $KCoFe(CN)_6 \cdot xH_2O$) was prepared using a coprecipitation route [1]. In detail, 0.1 mmol $K_3[Fe(CN)_6]$ and 0.7 g sodium dodecyl sulfate are dissolved in 20 mL deionized water. Then, 20 mL 1 mmol $Co(CH_3COO)_2 \cdot 4H_2O$ aqueous solution is slowly added into above mixture solution. After aging at room temperature for 24h, the resulting precipitates are collected by centrifugation and washed several times with deionized water. Finally, the products are freeze-dried for 48 h.

Calculation based on Nernst Equation

The electrochemical reaction of PT electrode in 1M HCl can be expressed as below:

$$PT + nH^+ + ne^- \rightleftharpoons H_nPT \qquad (1)$$

Nernst Equation:

$$\varphi = \varphi^\theta + \frac{2.303RT}{nF}\lg\frac{[PT][H^+]^n}{[H_nPT]} = \varphi^\theta + \frac{0.0592}{n}\lg\frac{[PT][H^+]^n}{[H_nPT]} \qquad (2)$$

Where φ is electrode potential; φ^θ is the standard potential; R is the universe gas constant: 8.314 J K^{-1} mol^{-1}; T is the temperature: 298.15 K; n is the electron transfer numbers; F is the Faraday constant: 96500 C mol^{-1}. The activity of solid PT and H_nPT is considered as 1, thus the equation can be further simplified as:

$$\varphi = \varphi^\theta + \frac{0.0592}{n}\lg[H^+]^n = \varphi^\theta + 0.0592\ \lg[H^+] \qquad (3)$$

Therefore, the potential difference of PT in a 1 M HCl solution (pH=1) and in a pH=7.8 aqueous solution (the pH value of 1M $CaCl_2$ solution is ~7.8) can be calculated as:

$$\Delta\varphi = 0.0592\ \lg\frac{[H^+]_1}{[H^+]_2}\ V = 0.0592\ \lg\frac{1}{10^{-7.8}}\ \mathrm{V} = 0.462\ \mathrm{V} \qquad (4)$$

Charge storage mechanism analysis

The employed characterization techniques and their conclusions are briefly categorized as follows:

(1) FTIR spectra revealed the conversion of carbonyl groups (C=O, 1668 cm^{-1}) to enolate groups ($C-O^-$, ~1460 cm^{-1}) ;

(2) XPS explorations strongly evidenced the reversible uptake of Ca^{2+} during discharging (355-340eV) and its removal after recharging. Meanwhile, XPS O *1s* spectra revealed the appearance of the C–O peak (530.3 eV) after discharging and its disappearance after charging.

(3) XRD results exhibit well-retained characteristic peaks, indicating the robust structural stability of PT materials during repeated cycling.

(4) SEM and TEM images clearly revealed the formation of large amounts of spheroidal particles as the discharging products, which are absent in the initial PT samples. These crystalline spheres consist of Ca, O, and C elements as revealed by EELS mapping analysis. For these unreacted PT materials, they show bulky morphology beneath these newly formed spheres and they are Ca-free, which is quite different from the reacted PT.

(5) In-depth XPS profiles were performed to examine the spatial difference by Ar ion cluster sputtering. After etching for 10 nm and 20 nm, the PT anode still exhibits featured in-depth XPS peaks of Ca2p, which clearly verify the storage of Ca in PT anodes.

(6) ICP-MS analysis is to quantify the content of Ca in the whole discharged PT anode, which is measured to be 16.9 g kg^{-1}, further support our assumption.

In summary, the above characterization results collectively support the 'chemical adsorption' mechanistic assumption where the Ca^{2+} cations adsorb to the negatively charged oxygen atoms upon electrochemical reduction of the carbonyl groups, and desorb reversibly during the reverse oxidation.

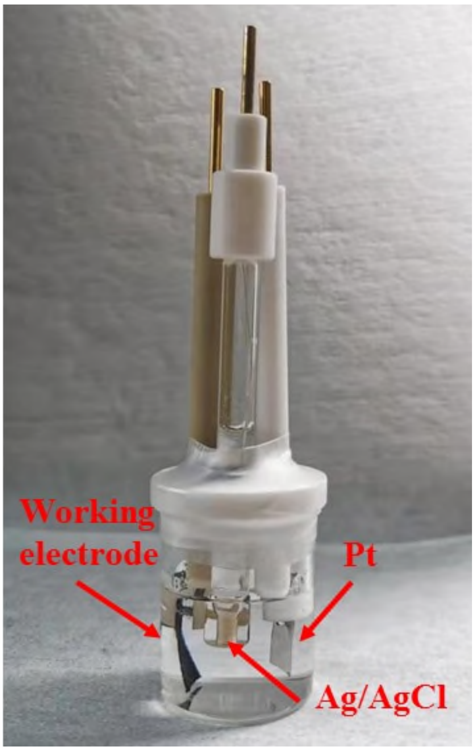

Supplementary Figure 1. Three electrode configuration for electrochemical performance test.

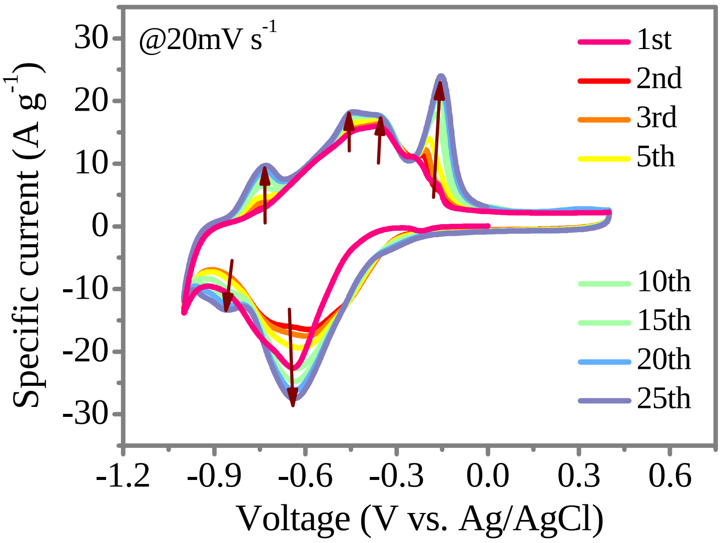

Supplementary Figure 2. CV profile of PT anode measured in degassed 1M $CaCl_2$ electrolyte at 20 mV s^{-1}, which only show a reduction peak at -0.63V vs. Ag/AgCl. Therefore, the reduction peak at -0.3V vs. Ag/AgCl in Figure 2a is ascribed to the reduction of dissolved O_2 in electrolyte and only present in the first cathodic scan.

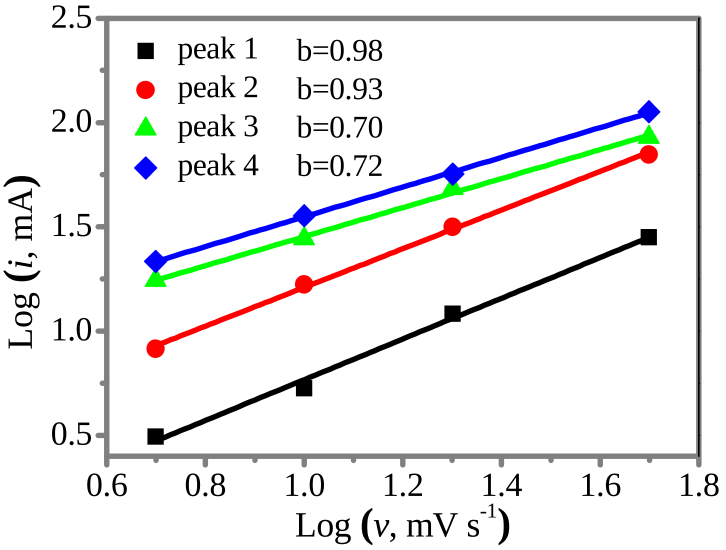

Supplementary Figure 3. Log(*i*) vs. Log(*v*) plots.

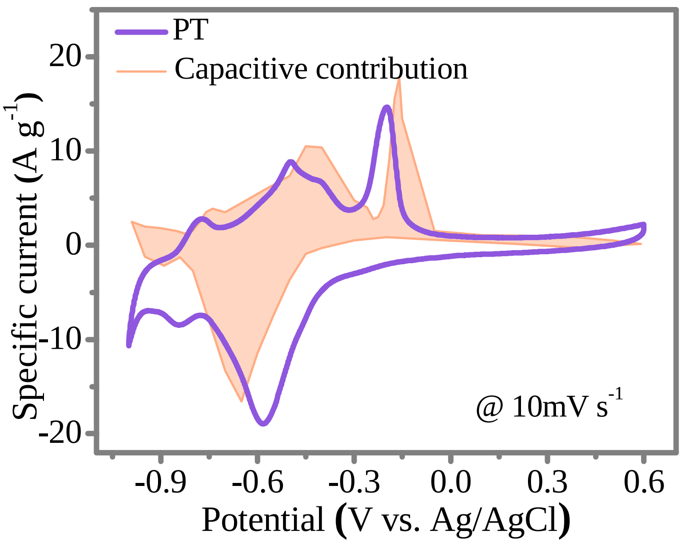

Supplementary Figure 4. The capacitive contribution to the total current of PT anode at 10 mV s^{-1}.

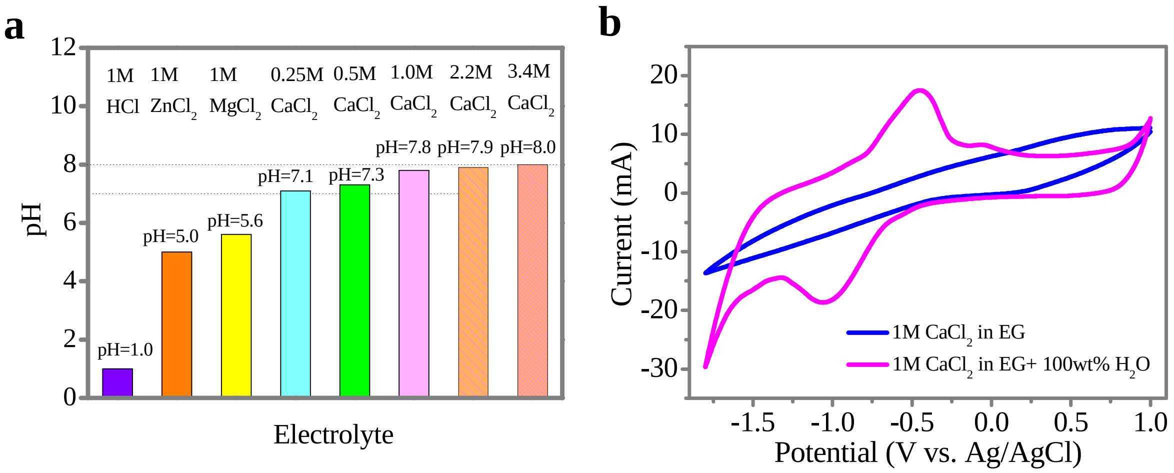

Supplementary Figure 5. **a** pH value of 1M HCl, 1M $ZnCl_2$, 1M $MgCl_2$ solution and aqueous $CaCl_2$ solution with different concentrations. **b** CV curves of the PT electrode in organic EG based electrolyte with and without adding H_2O.

As shown by the blue curve in Supplementary Figure 5b, the PT electrode demonstrates a very limited capacity (capacitor behavior) due to the absence of H^+ ions. For comparison, equal amount of H_2O was added into the EG-based non-aqueous electrolyte. As presented by the pink curve, a much larger capacity and obvious redox peaks are observed. From this result, we speculate that the insertion of H^+ exists during the storage of Ca^{2+} in PT electrode.

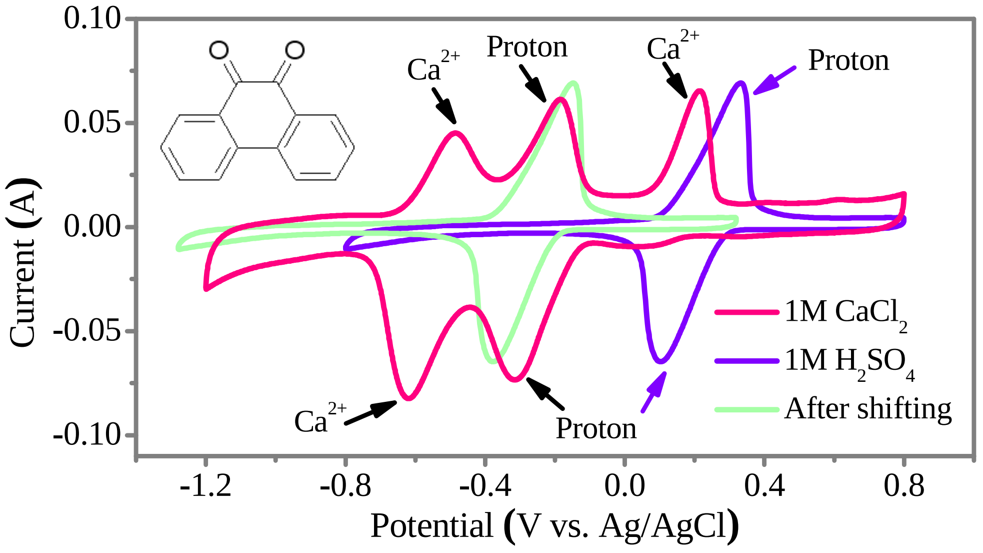

Supplementary Figure 6. CV curves of 9,10-PQ material in 1M $CaCl_2$ and 1M H_2SO_4 at 20 mV s^{-1}. The light green line represents corresponding CV curves in a pH=7.8 solution, calculated based on the Nernst equation.

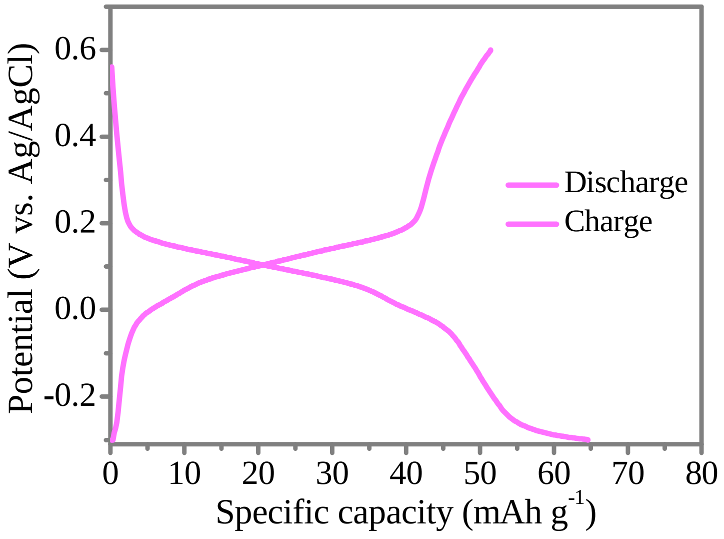

Supplementary Figure 7. GCD curve of PT electrode measured in 1M HCl electrolyte at 5 A g^{-1}.

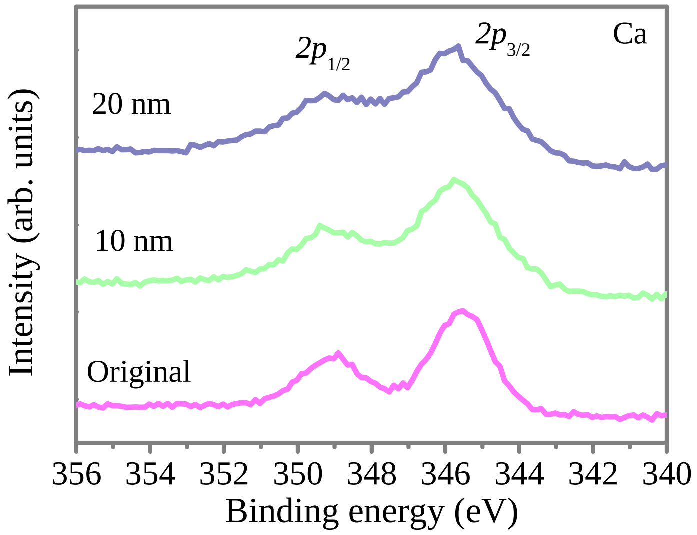

Supplementary Figure 8. In-depth XPS spectra of Ca *2p* on discharged PT electrode.

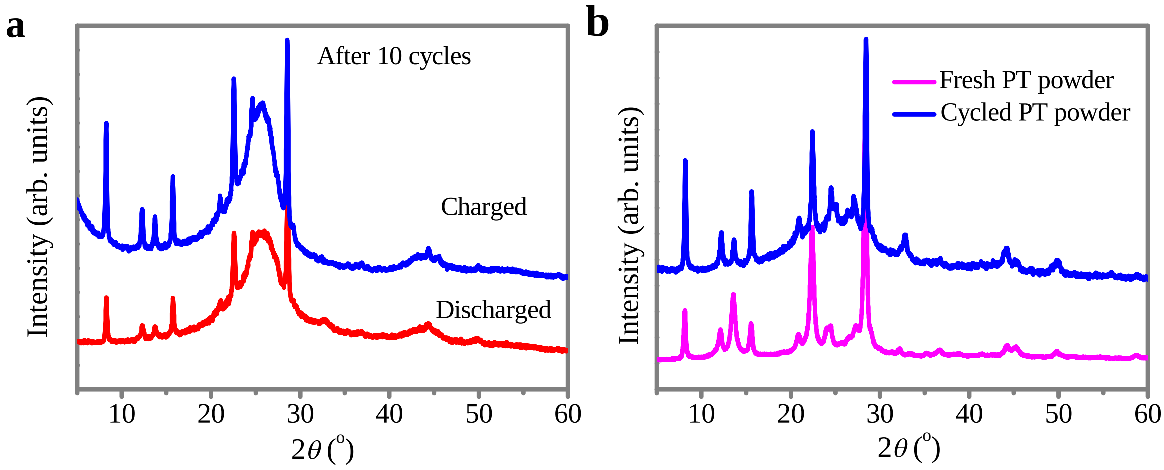

Supplementary Figure 9. **a** XRD profile of PT anode after 10 discharge/charge cycles at 0.1 A g^{-1}. **b** XRD profile of PT materials scratched from the carbon cloth substrate and its comparison with pristine and fresh PT powders.

To eliminate the influence of carbon cloth substrate, the electrode materials that are discharged to -0.8 V vs. Ag/AgCl after 10 cycles at 0.1 A g^{-1} are scratched from the carbon cloth substrate. The as-obtained XRD patterns are compared with pristine PT powder (Supplementary Figure 9b), indicating the robust structural stability of PT materials during repeated cycling.

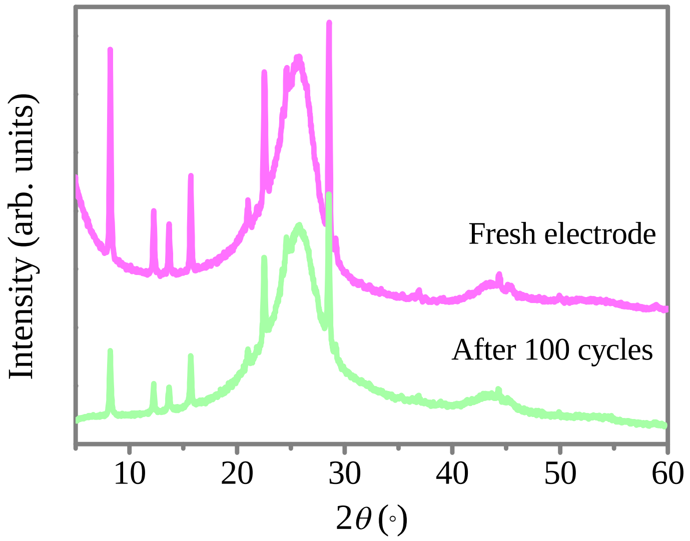

Supplementary Figure 10. XRD profile of PT electrode after cycling at 100 A g^{-1} for 100 cycles and its comparison with the fresh PT electrode.

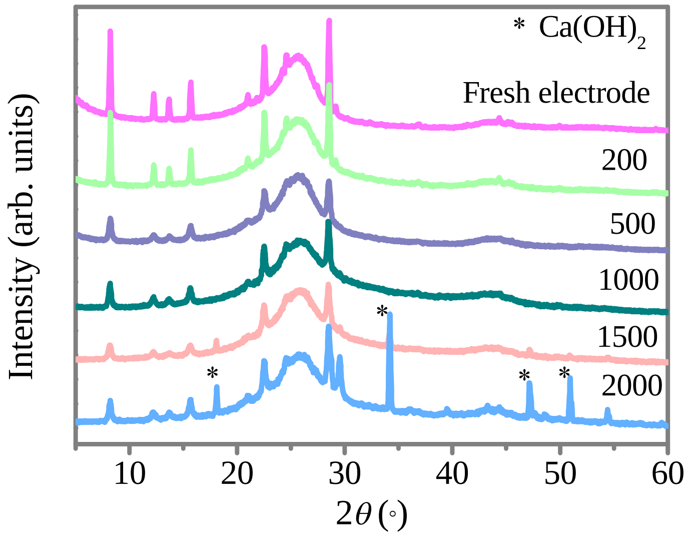

Supplementary Figure 11. XRD profile of PT electrode during cycling at 30 A g^{-1}.

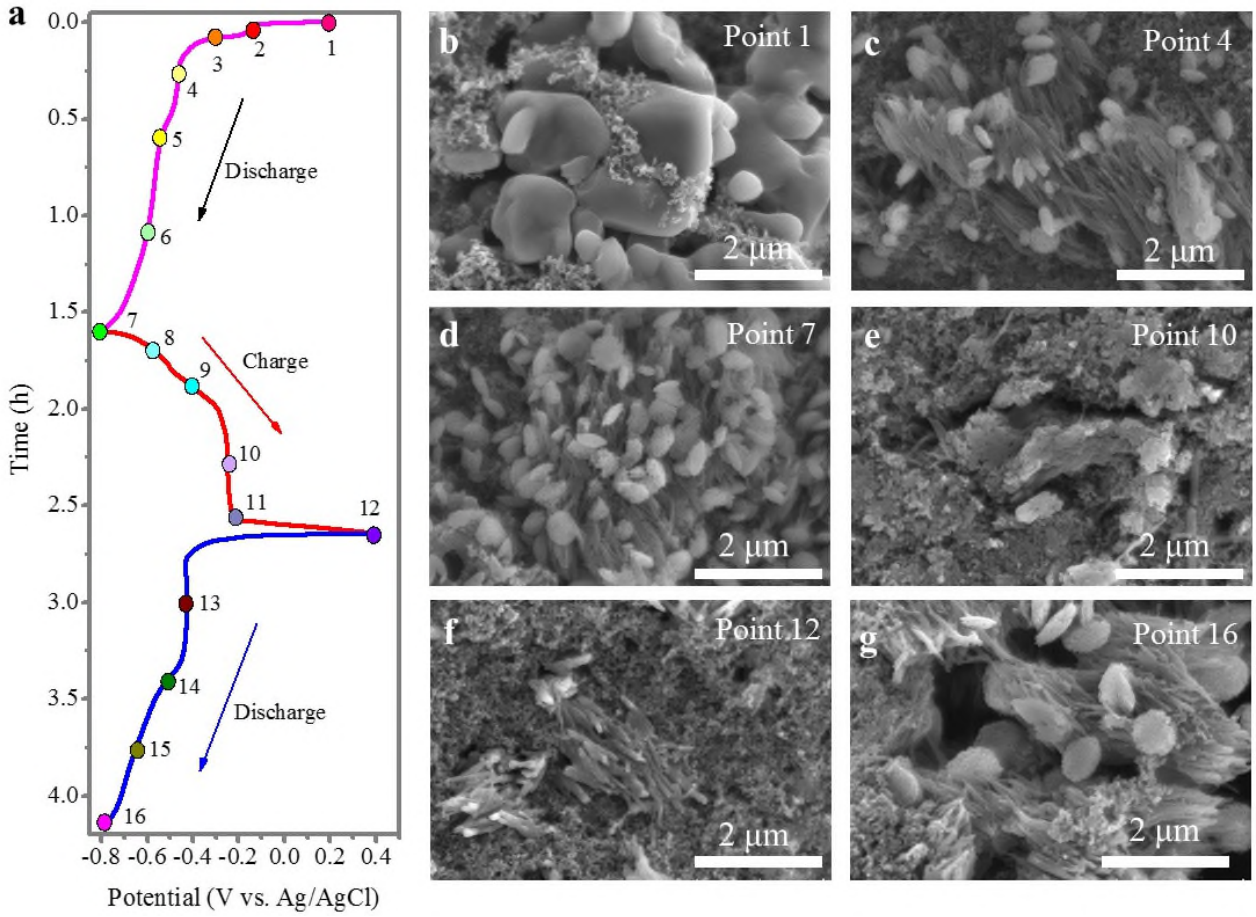

Supplementary Figure 12. **a** The GCD curve of PT electrode and **b-g** the corresponding morphology evolution measured at selected points.

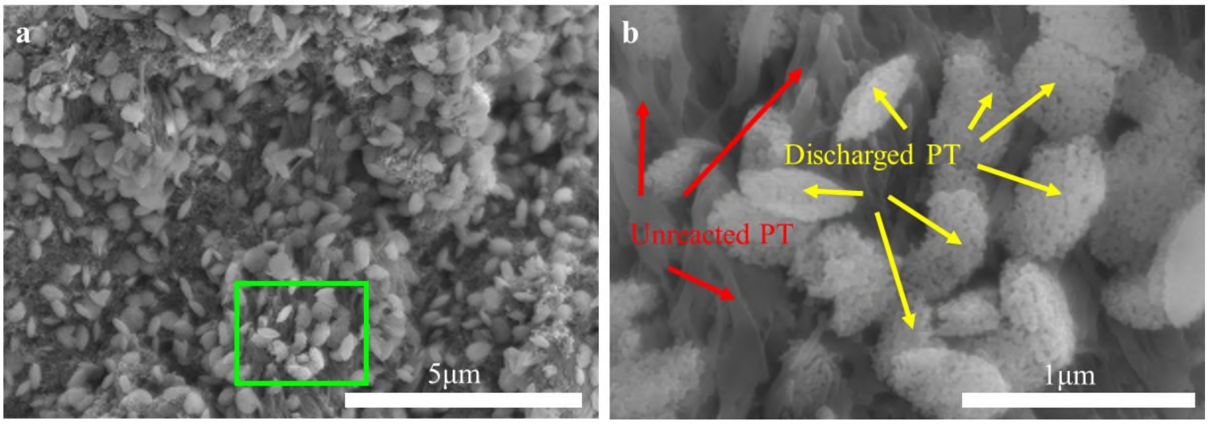

Supplementary Figure 13. **a** Low magnification and **b** high magnification SEM images of the discharged PT anode (point 7), which clearly reveals the formation of large amount of macrospheroids as the reaction products.

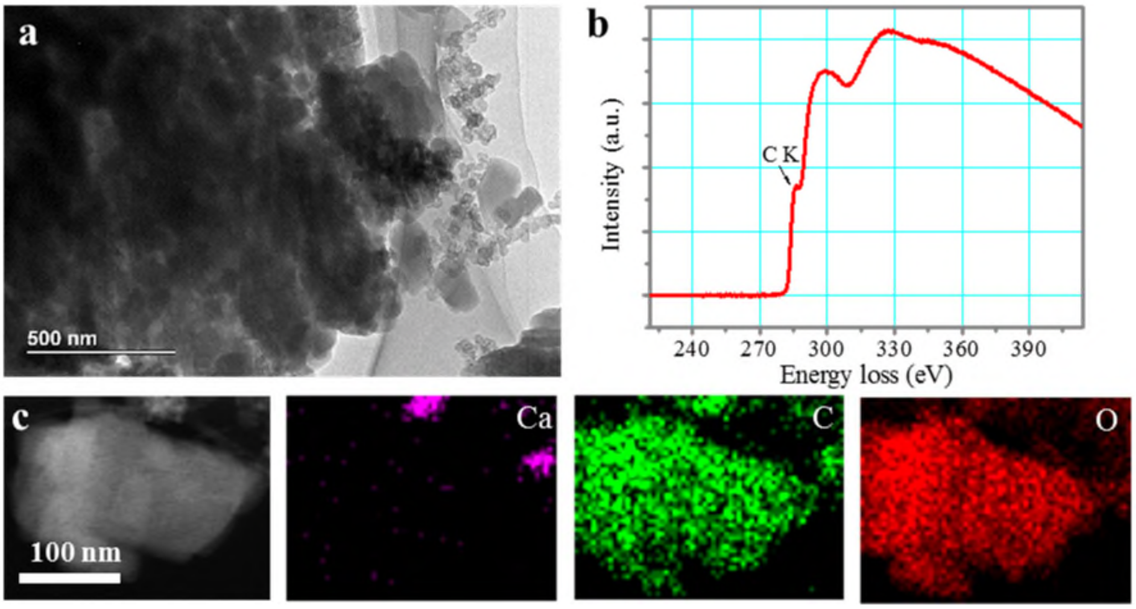

Supplementary Figure 14. TEM characterization on the unreacted PT component in the discharge PT electrode. **a** TEM image. **b** EELS spectrum. **c** EDS mapping image.

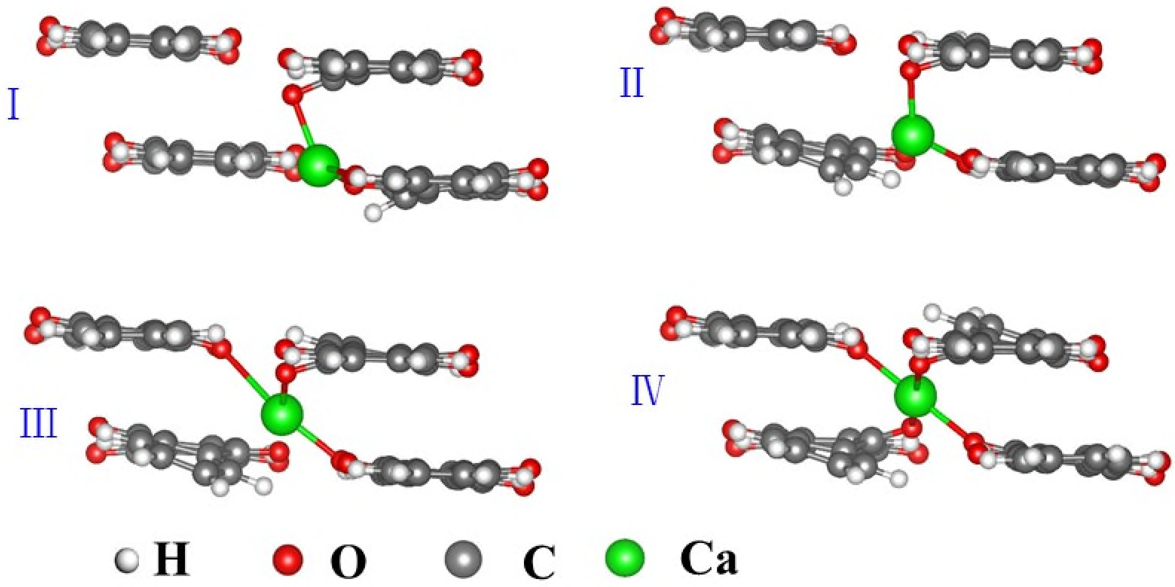

Supplementary Figure 15. Side view of representative equilibrium positions for a Ca ion inserted in the channel of PT crystal.

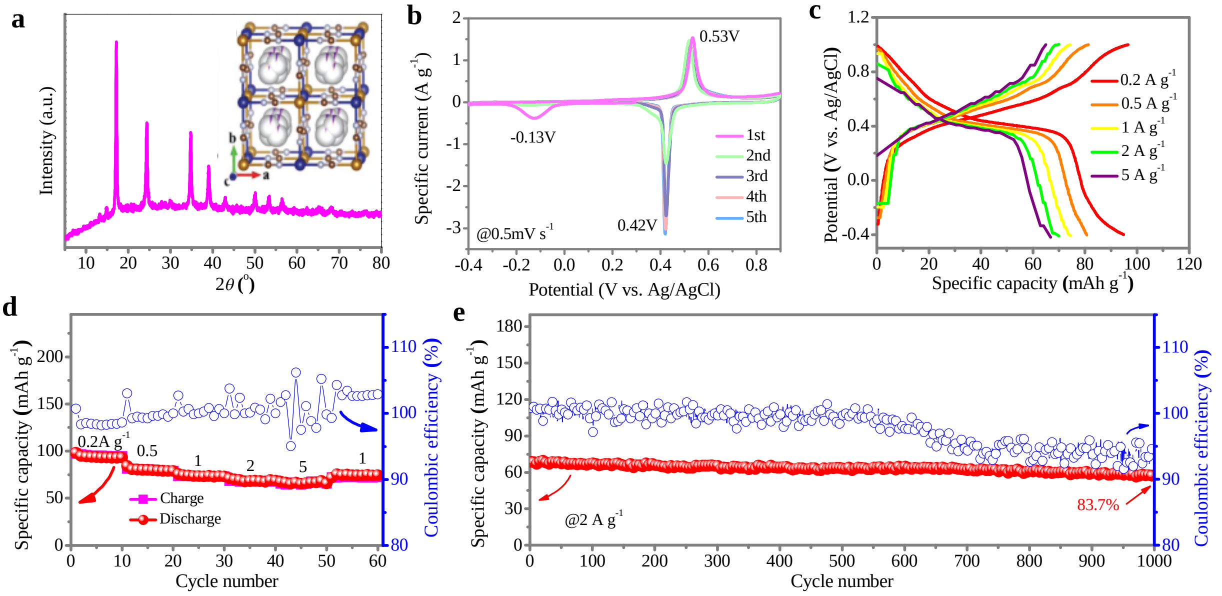

Supplementary Figure 16. Characterizations of KCoFe(CN)$_6$·xH$_2$O cathode. **a** XRD profile and corresponding crystal structure. **b** CV curves at 0.5 mV s^{-1}. **c** GCD curves at different specific currents. **d** Specific capacities at different specific currents. **e** Cycle performance at 2 A g^{-1} and the corresponding Coulombic efficiency during cycling.

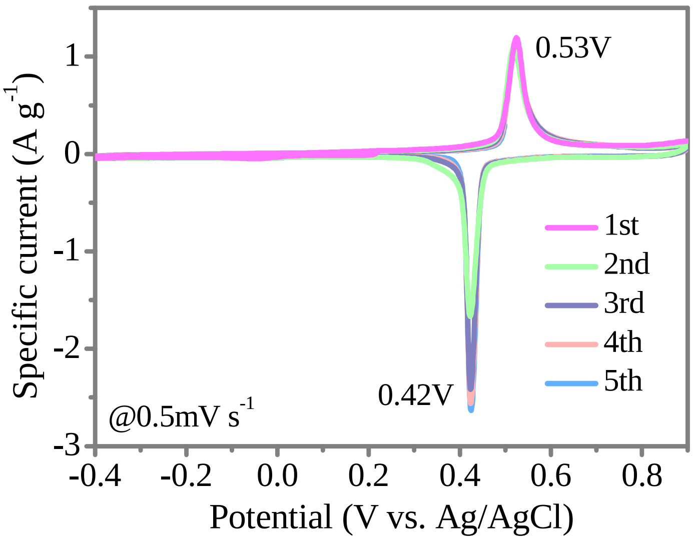

Supplementary Figure 17. The CV curve of KCoFe(CN)$_6$ cathode measured in degassed CaCl$_2$ electrolyte at 0.5 mVs^{-1}, signifying that the reduction peak at -0.13V vs. Ag/AgCl in Figure S17b is caused by the reduction of dissolved O$_2$ in aqueous electrolyte.

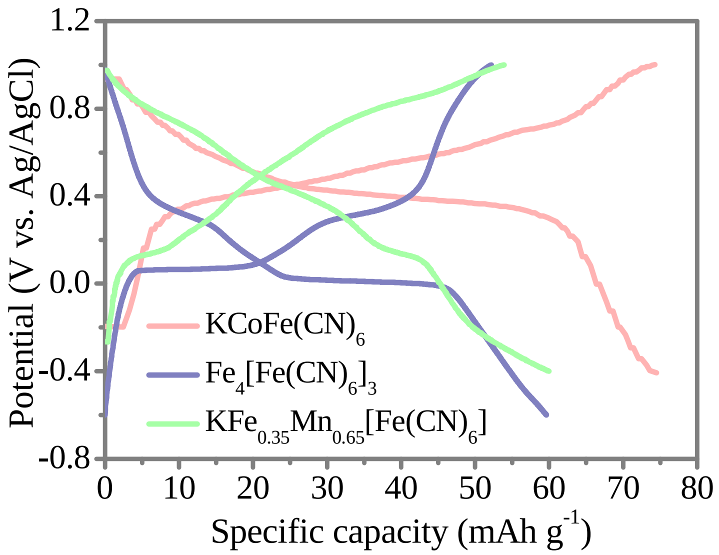

Supplementary Figure 18. The GCD curves of $KCoFe(CN)_6$, $Fe_4[Fe(CN)_6]_3$, and $KFe_{0.35}Mn_{0.65}Fe(CN)_6$ at 1 A g^{-1}.

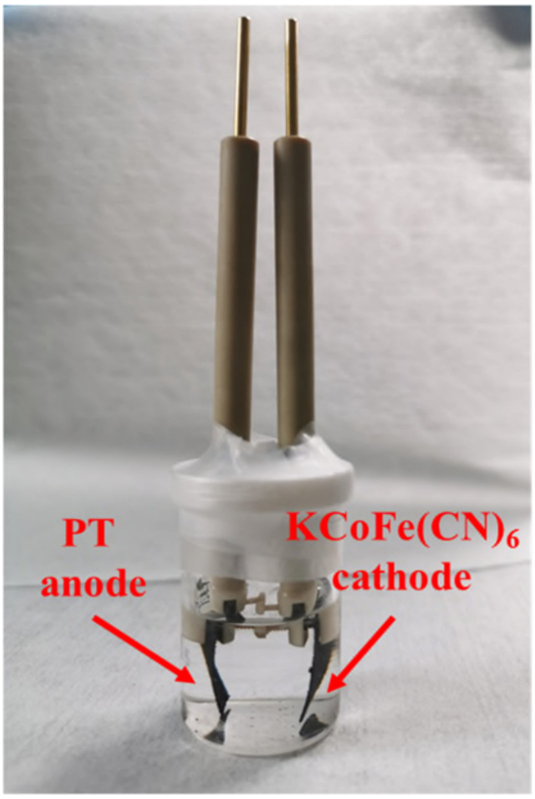

Supplementary Figure 19. Cell configuration of the PT// $KCoFe(CN)_6$ full cell.

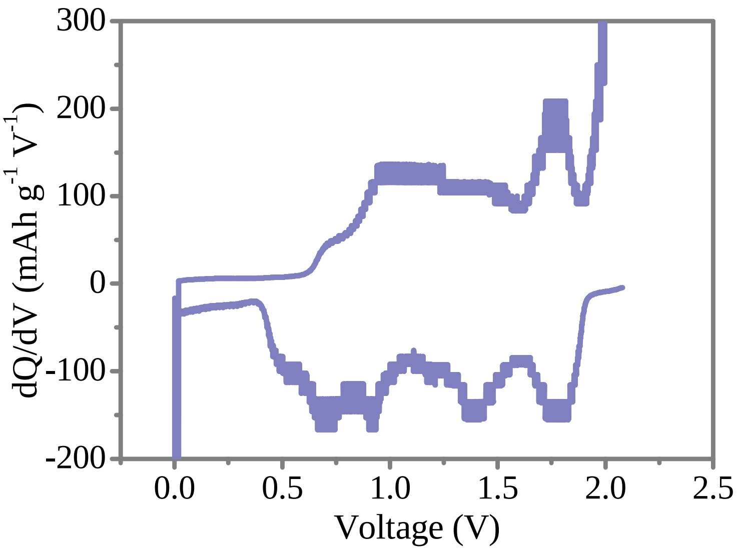

Supplementary Figure 20. The differential capacity (dQ/dV) curve of the full Ca-ion cell at 2 A g^{-1}.

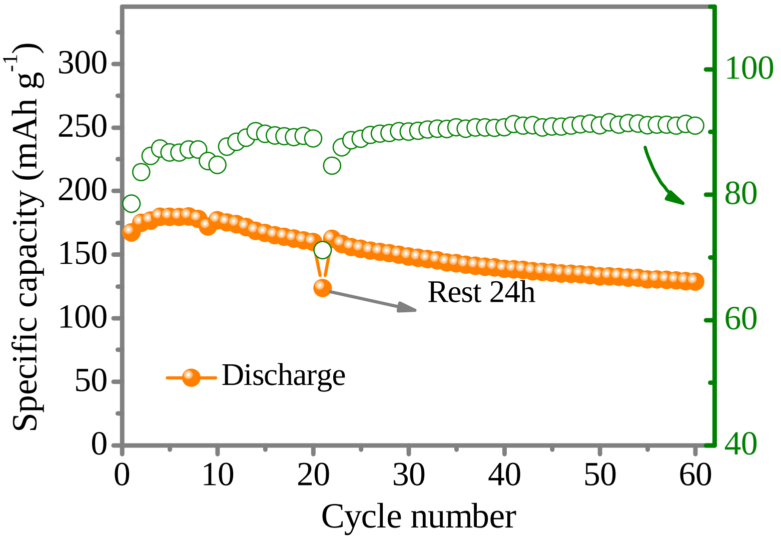

Supplementary Figure 21. Self-discharge performance of the full Ca-ion cell at 1 A g^{-1}.

Supplementary Table 1. Rietveld refined and theoretical calculated lattice parameters of crystalline phase of PT.

Lattice Parameters	a (nm)	b (nm)	c (nm)	α (°)	β (°)	γ (°)	V (nm^3)
Experimental	0.476	0.754	1.112	97.68	93.23	99.13	0.3890
Calculated	0.460	0.748	1.105	98.70	93.10	98.20	0.3703

Supplementary Table 2. Performance comparison with reported electrode materials for CIBs.

Configuration	Working Electrode	Counter electrode	Reference electrode	Electrolyte	Specific capacity @Specific current	Capacity retention/Cycle number/Specific current	Ref.
Three electrode system	PT	Pt	Ag/AgCl	1M $CaCl_2$ in water	150.5 mAh g^{-1}@5 A g^{-1} 86.1 mAh g^{-1} at 100 A g^{-1}	100%/3000/30 A g^{-1} (1C=317 mA g^{-1})	This work
	PNDIE	activated carbon (AC)	Ag/AgCl	2.5M $Ca(NO_3)_2$ in water	148 mAh g^{-1}@1C ~110 mAh g^{-1}@20C	~80%/4000/5C (1C=183 mA g^{-1})	2
	CaxCuHCF	Graphite foil	Ag/AgCl	2.5M $Ca(NO_3)_2$ in water	58.6 mAh g^{-1}@0.2C 42 mAh g^{-1}@12C	94%/1000/5C (1C = 60 mA g^{-1})	3
	$KNiFe(CN)_6$	Pt	Ag/AgCl	1M $Ca(NO_3)_2$ in water	50 mAh g^{-1}@0.2C 38.5 mAh g^{-1}@10C	/ (1C=50 mA g^{-1})	4
	$K_xNiFe(CN)_6$	AC	Ag/Ag^+	0.5 M $Ca(TFSI)_2$ in acetonitrile (AN)	50 mA h g^{-1} @ 25 µA cm^{-2}	/	5
Two electrode system	PT	$KCoFe(CN)_6$	/	1M $CaCl_2$ in water	179.5 mAh $g^{-1}{}_{PT}$@2 A g^{-1}	81.8%/3000/30 A g^{-1}	This work
	Sn	graphite	/	0.8 M $Ca(PF_6)_2$ in organic carbonates	72 mAh g^{-1}@0.1 A g^{-1} 40 mAh g^{-1}@0.4 A g^{-1}	95%/200~350/0.1A g^{-1}	6
	Sn	AC	/	0.8 M $Ca(PF_6)_2$ in organic carbonates	92 mAh g^{-1}@ 0.1 A g^{-1} 62 mAh g^{-1}@1.6 A g^{-1}	84%/1000/0.2 A g^{-1}	7
	Graphite	Ca metal	/	0.5M $Ca(BH_4)_2$ in dimethylacetamide	89 mAh g^{-1}@0.05A g^{-1} 67 mAh g^{-1}@2 A g^{-1}	95%/40/0.1 A g^{-1}	8
	PTCDA	AC	/	Saturated $Ca(NO_3)_2$ in water	87 mAh g^{-1}@0.02 A g^{-1}	/	9
	$KFeFe(CN)_6$	Ni-based metal-organic compound	/	1 M $Ca(ClO_4)_2$ in CH_3CN	82 mAh g^{-1}@0.1 A g^{-1}	62%/100/0.1 A g^{-1}	10
	$Mg_{0.25}V_2O_5 \cdot H_2O$	Activated carbon cloth (ACC)	/	0.8 M $Ca(TFSI)_2$ in organic carbonates	120 mAh g^{-1}@0.02A g^{-1} 70.2 mAh g^{-1}@0.1A g^{-1}	86.9%/500/0.1A g^{-1}	11

	$NH_4V_4O_{10}$	manganese 2-aminoterephthalate (Mn-bdcNH$_2$)	/	$Ca(ClO_4)_2 \cdot xH_2O$ in AN	75 mAh g^{-1} @0.1 A g^{-1}	~100%/100/0.1A g^{-1}	12

Supplementary Table 3. ICP-MS analysis on the content of Ca in discharged PT electrode.

Sample	V_0 (mL)	Element	Content (mg kg^{-1})	Content (%)
Discharge PT anode	25	Ca	16932.4	1.7

Supplementary References

1 Ma, L. *et al.* Achieving High-Voltage and High-Capacity Aqueous Rechargeable Zinc Ion Battery by Incorporating Two-Species Redox Reaction. *Adv. Energy Mater.* **9**, 1902446 (2019).

2 Gheytani, S. *et al.* An Aqueous Ca-Ion Battery. *Adv. Sci.* **4**, 1700465 (2017).

3 Adil, M. *et al.* Practical Aqueous Calcium-Ion Battery Full-Cells for Future Stationary Storage. *Acs Appl. Mater. Interfaces* **12**, 11489-11503 (2020).

4 Wang, R. Y., Wessells, C. D., Huggins, R. A. & Cui, Y. Highly Reversible Open Framework Nanoscale Electrodes for Divalent Ion Batteries. *Nano Lett.* **13**, 5748-5752 (2013).

5 Tojo, T., Sugiura, Y., Inada, R. & Sakurai, Y. Reversible Calcium Ion Batteries Using a Dehydrated Prussian Blue Analogue Cathode. *Electrochim. Acta* **207**, 22-27 (2016).

6 Wang, M. *et al.* Reversible calcium alloying enables a practical room-temperature rechargeable calcium-ion battery with a high discharge voltage. *Nat. Chem.* **10**, 667-672 (2018).

7 Wu, N. *et al.* A Calcium-Ion Hybrid Energy Storage Device with High Capacity and Long Cycling Life under Room Temperature. *Adv. Energy Mater.* **9**, 1803865 (2019).

8 Park, J. *et al.* Stable and High-Power Calcium-Ion Batteries Enabled by Calcium Intercalation into Graphite. *Adv. Mater.* **32**, 1904411 (2020).

9 Rodríguez-Pérez, I. A. *et al.* Mg-Ion Battery Electrode: An Organic Solid's Herringbone Structure Squeezed upon Mg-Ion Insertion. *J. Am. Chem. Soc.* **139**, 13031-13037 (2017).

10 Vo, T. N., Hur, J. & Kim, I. T. Enabling High Performance Calcium-Ion Batteries from Prussian Blue and Metal–Organic Compound Materials. *ACS Sustain. Chem. Eng.* **8**, 2596-2601 (2020).

11 Xu, X. *et al.* Bilayered $Mg_{0.25}V_2O_5 \cdot H_2O$ as a Stable Cathode for Rechargeable Ca-Ion Batteries. *ACS Energy Lett.* **4**, 1328-1335 (2019).

12 Vo, T. N., Kim, H., Hur, J., Choi, W. & Kim, I. T. Surfactant-assisted ammonium vanadium oxide as a superior cathode for calcium-ion batteries. *J. Mater. Chem. A* **6**, 22645-22654 (2018).
