# Supplementary material for: Proton-assisted calcium-ion storage in aromatic organic molecular crystal with coplanar stacked structure
Source: Nat Commun. 2021 Apr 23;12:2400. doi: 10.1038/s41467-021-22698-9 (PMC8065044; doi:10.1038/s41467-021-22698-9)
Supplement: Supplementary file 3 — Descriptions of Additional Supplementary Files [file 41467_2021_22698_MOESM3_ESM.pdf]

Descriptions of Additional Supplementary Files

Supplementary Movie 1
Description: The migration pathway of Ca^{2+} within the PT crystal.
